# The eco-epidemiology of *Triatoma infestans* in the temperate Monte Desert ecoregion of mid-western Argentina

**DOI:** 10.1590/0074-02760160519

**Published:** 2017-10

**Authors:** Ana Laura Carbajal-de-la-Fuente, Yael Mariana Provecho, María del Pilar Fernández, Marta Victoria Cardinal, Patricia Lencina, Cynthia Spillmann, Ricardo Esteban Gürtler

**Affiliations:** 1 University of Buenos Aires, Institute of Ecology, Genetics and Evolution of Buenos Aires, Faculty of Exact and Natural Sciences, National Scientific and Technical Research Council, Ciudad Autónoma de Buenos Aires, Argentina; 2 Health Ministry, Division of Zoonoses, Vectors and Reservoirs, Mendoza, Argentina; 3 Health Ministry, Laboratory of Public Health, Mendoza, Argentina; 4 National Health Ministry, National Chagas Program, Córdoba, Argentina

**Keywords:** Triatoma infestans, Chagas disease, eco-epidemiology, Monte Desert ecoregion

## Abstract

**BACKGROUND:**

The eco-epidemiological status of Chagas disease in the Monte Desert ecoregion of western Argentina is largely unknown. We investigated the environmental and socio-demographic determinants of house infestation with *Triatoma infestans*, bug abundance, vector infection with *Trypanosoma cruzi* and host-feeding sources in a well-defined rural area of Lavalle Department in the Mendoza province.

**METHODS:**

Technical personnel inspected 198 houses for evidence of infestation with *T. infestans*, and the 76 houses included in the current study were re-inspected. In parallel with the vector survey, an environmental and socio-demographic survey was also conducted. Univariate risk factor analysis for domiciliary infestation was carried out using Firth penalised logistic regression. We fitted generalised linear models for house infestation and bug abundance. Blood meals were tested with a direct ELISA assay, and *T. cruzi* infection was determined using a hot-start polymerase chain reaction (PCR) targeting the kinetoplast minicircle (kDNA-PCR).

**FINDINGS:**

The households studied included an aged population living in precarious houses whose main economic activities included goat husbandry. *T. infestans* was found in 21.2% of 198 houses and in 55.3% of the 76 re-inspected houses. Peridomestic habitats exhibited higher infestation rates and bug abundances than did domiciles, and goat corrals showed high levels of infestation. The main host-feeding sources were goats. Vector infection was present in 10.2% of domiciles and 3.2% of peridomiciles. Generalised linear models showed that peridomestic infestation was positively and significantly associated with the presence of mud walls and the abundance of chickens and goats, and bug abundance increased with the number of all hosts except rabbits.

**MAIN CONCLUSIONS:**

We highlight the relative importance of specific peridomestic structures (i.e., goat corrals and chicken coops) associated with construction materials and host abundance as sources of persistent bug infestation driving domestic colonisation. Environmental management strategies framed in a community-based programme combined with improved insecticide spraying and sustained vector surveillance are needed to effectively suppress local *T. infestans* populations.

Chagas disease ranks highly among the main neglected tropical diseases in the Americas (Hotez 2014). The main vector for Chagas disease, *Triatoma infestans* (Klug, 1834) (Hemiptera: Reduviidae), has historically played a crucial role in the Southern Cone Region. Although the geographical range of *T. infestans* has been strongly reduced over the last three decades, this species still persists in the Gran Chaco eco-region of Argentina, Bolivia and Paraguay, where it represents a serious health problem (Hotez 2014). Chagas disease affects approximately 1.6 million people in Argentina, and *T. infestans* is present in a large fraction of the territory (WHO 2015).

Chagas disease in the Argentine Chaco is characterised by a complex eco-epidemiological scenario with high levels of house infestations by *T. infestans* (Gaspe et al. 2015) and increasing professional vector control efforts over the last decade (MSAL 2016). Several efforts have been made to understand the processes related to *T. infestans* house infestation and re-infestation patterns as well as the transmission of *Trypanosoma cruzi* (Chagas, 1909) (Gürtler et al. 2007). However, the eco-epidemiology of Chagas disease remains mostly unknown in western Argentina, where the SW extreme of the Gran Chaco creates a biogeographic transition area known as the Monte Desert (Roig et al. 2009). Mendoza province is located at the heart of the Monte Desert ecoregion. As in other endemic areas, political and economic decisions have affected the activities of the local vector control programme, generating periods when insecticide spraying was conducted in a pulsed fashion followed by years of total inactivity (Sosa-Estani et al. 2006). As part of a controversial decentralisation process, vector control activities were transferred to Mendoza province in 1982, at which point vector control notoriously declined (Sosa-Estani et al. 2006). Argentina’s National Chagas Program has classified Mendoza as a high-risk area on the basis of high *T. cruzi* seroprevalence in vulnerable populations and increasing domiciliary infestation since 2008 (MSAL 2016).

Mendoza province shows two clearly differentiated regions: irrigated dry lands (oases) and non-irrigated dry lands (desert) (Abraham & Torres 2014). The latter environment prevails in Lavalle Department (Montaña et al. 2005) where the local population has been economically and socio-politically marginalised (Cepparo & Torres 2015). Rural poverty and land occupation patterns have been associated with *T. infestans* house infestations and the transmission of Chagas disease (Briceño-León 2009).

In an effort to improve vector control activities, we conducted a randomised intervention trial to evaluate the efficiency and effectiveness of insecticide spraying operations in Lavalle Department (Carbajal-de-la-Fuente et al. 2017) as part of a wider study on the eco-epidemiology and control of *T. infestans* in the region. The aims of the current study were to assess the environmental and socio-demographic determinants of house infestation with *T. infestans*, bug abundance, vector infection with *T. cruzi* and its host-feeding patterns in a well-defined rural area of Lavalle Department.

## MATERIALS AND METHODS

**Fig. 1:**
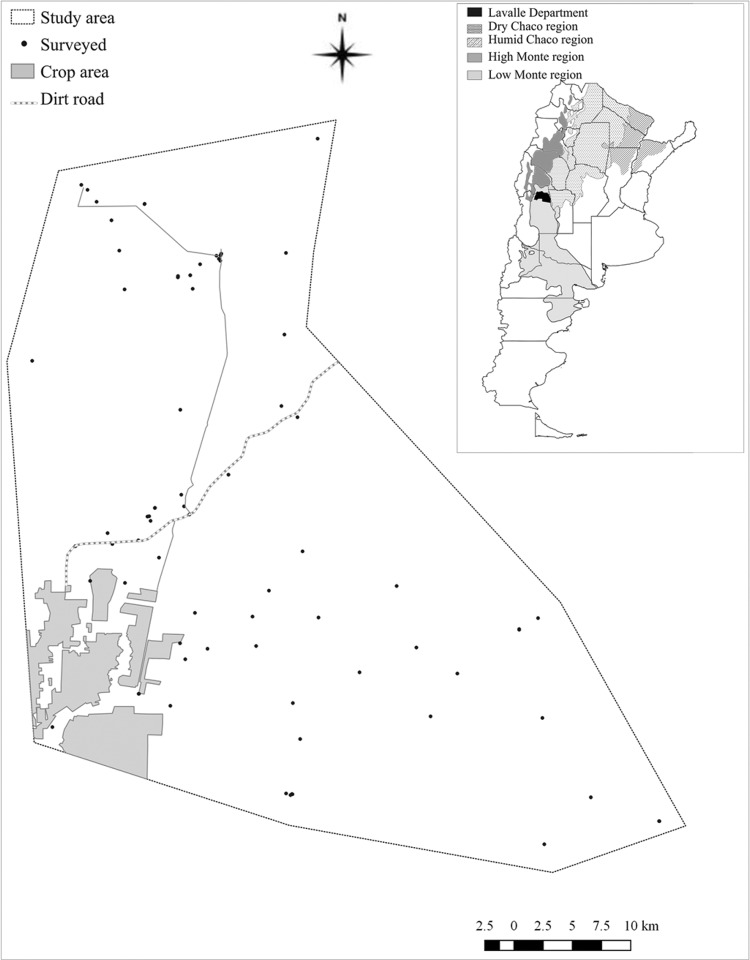
: map of the study area showing the location of the 76 study houses in the La Asunción and San José Districts, Mendoza, Argentina.

*Study area* - Fieldwork was conducted in contiguous rural sections of La Asunción, San José, and the surrounding area, Lavalle Department (32º 29.731 S, 68º 09.467 W), in Mendoza province, Argentina, during April-May 2013 (Fig. 1). According to the 2010 census records, 973 people inhabited the study area (http://www.deie.mendoza.gov.ar/#). According to Mendoza’s Chagas disease control programme records, vector control activities in the study area had historically been very sparse, and the last insecticide spraying campaign had been carried out between two and 10 years before this study, depending on house accessibility. The selection of the study area considered the last community-wide insecticide spraying registered and the fact that preliminary evidence of house infestation ranged from 30-40%. The study area belongs to the Monte (scrubland) biogeographic province (Roig et al. 2009). This is a wide plain slightly sloping northeast with dunes and saline soils, a semiarid climate and xerophytic vegetation. Local meteorological data were obtained from *Telteca* station. The annual mean temperature was 17.8ºC (mean minimum and maximum, 8.7 and 26.7ºC), and the total precipitation was 172.2 mm in 2013.

*Study design and vector survey* - As part of a randomised intervention trial evaluating the efficiency and effectiveness of insecticide spraying operations, an exploratory survey of house infestation was undertaken in April 2013 (Carbajal-de-la-Fuente et al. 2017). Experienced field personnel of the National Vector Control Program (NVCP) from Mendoza inspected 198 houses for evidence of current or past infestation (i.e., live triatomines, eggs and faeces). Houses where evidence of infestation was found (n = 76) were included in the current study and re-inspected in May 2013 before spraying operations began. All of the 198 houses were sprayed with pyrethroid insecticides during May-June 2013 (Carbajal-de-la-Fuente et al. 2017). The 76 houses included in this study were scattered over 1,400 km^2^, with distances between houses ranging from a few hundred meters up to 50 km. The housing compounds included human sleeping quarters (i.e., domiciles) and peridomestic annexes, such as goat corrals, chicken coops, storerooms and other structures (excluding latrines), regardless of the distances to human sleeping quarters.

Fieldwork was conducted by four teams of three people each, which included two skilled bug collectors from the NVCP who searched for triatomines and sprayed insecticides and a third person who recorded environmental and demographic data. In each house, the study aims and project phases were explained to an adult inhabitant who also provided oral consent to access the premises. Each house was identified with a sticker and geo-referenced with a Global Positioning System (GPS) receiver (Garmin Legend). All domiciles and peridomestic sites were inspected for triatomines by two people using timed manual collections (TMCs) and an aerosol spray (0.2% tetramethrin, Espacial, Reopen, Buenos Aires, Argentina) for 30 min.

The collected bugs were stored in plastic bags labelled with the house number and specific bug collection site and transported to the field laboratory, where they were identified and counted according to species, stage or sex. To identify bloodmeal sources, a random sample corresponding to 28% (n = 415) of all collected third- to fifth-instar nymphs and adult bugs were dissected, and the midgut bloodmeal contents were extracted into a previously labelled and weighed vial (Gürtler et al. 2014). The blood meal were tested with a direct ELISA assay against human, dog, cat, chicken, pig, goat, rabbit, murid rodent (rat or mouse) and Caviidae rodent (cavies) antisera with high sensitivities and specificities as previously described (Gürtler et al. 2014). We reported the proportion of reactive bugs (i.e., those positive against any of the tested antisera) that contained each type of host blood.

For molecular diagnosis of *T. cruzi* infection, each bug was dissected, and the rectal ampoule (n = 407) was separated into a labelled vial containing 50 µL of sterile water. Rectal contents were boiled for 15 min, and DNA was extracted using DNAzol® (Invitrogen, USA) as described (Marcet et al. 2006). Positive (i.e., contents from the rectal ampoule of a bug infected with *T. cruzi* as determined by optic microscopy) and negative controls (sterile water or contents of the rectal ampoule of a bug that only fed on pigeons) were included in each round of DNA extraction. *T. cruzi* infection was determined using a hot-start polymerase chain reaction (PCR) targeting the kinetoplast minicircle (kDNA-PCR) and Taq Platinum DNA polymerase (Invitrogen, USA) following standardised protocols (Burgos et al. 2005). Each PCR included a positive control (DNA from a *T. cruzi* culture) and two negative controls, one for controlling the reagent mixture and one for the DNA loading procedure, using water instead of DNA. Eight insects for which the rectal ampoule could not be extracted were excluded.

*Environmental and socio-demographic survey* - In parallel with the vector survey, the following information was collected from an adult household member: full name of the head of the household; sex; place of birth; years of residence in the house; the number of residents by age group (0-5, 6-14, and 15 or more years of age). A sketch map of the spatial location of all structures in each house compound was drawn, and each structure was given a unique code according to its main use. The distance to sylvatic habitats was registered. For each site at every house compound, we recorded the building materials used in the walls and roofs. Specifically, for domiciles, the degree of wall deterioration (“cracked walls”, an ordinal variable scored among one of five levels ranging from few to abundant cracks, as determined visually), presence of window screens (wire mesh), use of domestic insecticides (type, frequency, purpose), source of light and presence of animals (dogs, cats or chicken) sleeping indoors were also registered. For peridomestic sites, the number of domestic animals of each type (dogs, cats, poultry, rabbits, goats, pigs, cows, and equines) and refuge availability for *T. infestans* were registered for each site. The latter was determined visually by a skilled member of the research team and scored among one of five levels ranging from absent to very abundant refuges as described elsewhere (Gurevitz et al. 2011). Each site was classified into ecotopes (type of habitat) according to their construction characteristics and use.

*Data analysis* - The prevalence of house infestation was calculated as the proportion of all houses inspected where at least one *T. infestans* was found in any (domestic or peridomestic) site by timed-manual searches. House infestation refers to the finding of at least one *T. infestans* in any domestic or peridomestic site of houses re-inspected for infestation (n = 76). The term “house infestation prevalence” was defined as the number of infested houses (in the sample of houses re-inspected) relative to the total study houses (n = 198) and likely represents the lower bounds of house infestation prevalence in the area. Thereafter, the site-specific infestation prevalence was defined relative to the 76 re-inspected houses. To standardise the catch per unit-effort indices given the variable number of inspected sites across house compounds, the total search effort per house compound was divided by the number of inspected sites to estimate the search time per inspected site; then, all site-specific bug abundances were scaled to 15 min/person. Agresti-Coull binomial 95% confidence intervals (95% CIs) were used for house infestation, infection prevalence, and the proportion of bugs with given bloodmeal sources (Brown et al. 2001).

Domestic and peridomestic infestations were assessed separately, because field observations and exploratory analyses suggested that infestation occurred mainly in peridomiciles and that bugs eventually invaded human sleeping quarters (i.e., domiciles). Therefore, the explanatory variables considered as potential risk factors for domiciliary infestation included construction characteristics (wall and roof building materials and cracked walls), the presence of domestic animals resting indoors, household use of domestic insecticides as well as the type and site of application for those chemicals, window screens, and source of light. Household wealth was measured by the goat-equivalent index, which uses a small stock unit to quantify the total number of livestock (cows, pigs, and goats) and poultry owned by each household in terms of goat biomass (Gaspe et al. 2015). Univariate risk factor analysis for domiciliary infestation was carried out employing the Firth penalised logistic regression implemented in Stata statistical software, release 12.0. (Stata Corp, College Station, Texas, USA). The Firth penalised logistic regression produces finite, consistent estimates of regression parameters when the maximum likelihood estimates do not exist because of complete or quasi-complete data separation and reduces small-sample bias (Heinze & Schemper 2002).

Second, we analysed infestation risk depending on type of habitat (ecotope), including domiciles, and assessed the effect of the housing compound as a random variable (infested sites are usually aggregated at house compound level) via a generalised linear model with mixed effects and logit as the link function (GLMM) implemented in R 2.7.0 with the R-package “lme4” (Bates et al. 2009). Specifically for peridomestic sites, a multivariate risk factor analysis of infestation and bug abundance was carried out using generalised linear models with logit and log as the link functions, respectively. The models included 321 peridomestic sites. We used a multimodel inference approach based on Akaike’s information criterion to estimate the model-averaged effect size [odds ratio (OR), or incidence rate ratio (IRR)] and relative importance (RI) given the variables and models considered (Burnham & Anderson 2003) using the R-package “MuMin” (Barton 2009) as described by Gaspe et al. (2015). The RI of each variable was defined as the sum of Akaike weights in each model in which the variable was present (Burnham & Anderson 2003). The explanatory variables considered were host abundance (dog or cat, chicken, goat, cow or horse and rabbit) and construction materials. In the case of chicken, goat and cow or horse abundance, host counts were rescaled to tens of individuals. Missing values were assumed to occur completely at random and represented 1.2% of the data in only one variable (construction material). List-wise deletion was employed in order to use a multi-model inference approach as suggested by Burnham and Anderson (2003).

The infestation model employed was a logistic regression, and the bug abundance model was a negative binomial model (due to overdispersion of bug abundance), which included the natural-log (minutes-person) as an offset because the unit of catch effort differed among sites. For bug abundance, Poisson regression models were also assessed.

Multicolinearity was assessed using the variance inflation factor (VIF), and model fitting for the logistic regression was assessed with the Hosmer-Lemeshow goodness of fit test and the receiver operator curve (ROC) implemented in R 2.7.0 as described elsewhere (Gaspe et al. 2015). To assess model sensitivity and specificity, we employed an optimal threshold value that minimised the sum of the error frequencies (Schisterman et al. 2005). This value was obtained by finding the maximum sensitivity and specificity sum for all threshold values t (sens(t)+spec(t)) using the pROC R-package (Robin et al. 2011). Additionally, the H-index was employed as an alternative measure of the model classification performance (Hand 2009). This aggregated index of performance takes into account misclassification costs, which seek to quantify the relative severity of one type of error over another. In these models, we considered that misclassifying an infested site as non-infested was a greater (more costly) mistake than misclassifying a non-infested site. Higher H-index indicated better performance. The H-index allows comparisons of models across different datasets and classifiers and was implemented using the R-package “hmeasure” (Hand 2009).

## RESULTS

At least one *T. infestans* was found in 42 (55.3%) of 76 re-inspected houses, which yields a house infestation prevalence of 21.2% in the total study houses (n = 198). Among the 76 re-inspected houses, peridomestic sites (n = 321) exhibited a significantly higher frequency of infestation (51.3%, IC = 40.3%-62.2%) than did domestic sites (13.2%, IC = 7.1%-22.7%).

Domestic infestation was significantly and positively related to peridomestic infestation (Fisher’s exact test: p < 0.001). A large number of adult and triatomine nymphs (n = 1,686) were collected from domestic and peridomestic sites. Most (93.2%) *T. infestans* (1,131 nymphs and 441 adults) were collected in chicken coops and goat corrals, whereas the remainder (6.8%, 63 nymphs and 51 adults) were collected at domestic sites. A kennel with four dogs adjacent to a chicken coop harboured 708 *T. infestans* (Table II). Eleven adult and nymphal specimens of *Triatoma platensis,* Neiva 1913 were captured in chicken coops from two houses.

**TABLE II:** Factors associated with site-specific infestation and the abundance of *Triatoma infestans* in 76 domiciles in the La Asunción and San José districts, Mendoza, May 2013

	Variable	Infestation prevalence^a^	RI	OR (CI_95_)	p	Median bug abundance^b^	RI	IRR (CI_95_)	p
	
		(CI_95_, nº of sites )				(1st-3rd quartiles, nº of infested sites)			
Model 1	Intercept			0.3 (0.2-0.5)	< 0.001**			3.2 (1.8-6)	0.001**
	Ecotope								
	Goat corrals	23.0 (16.0-34.0, 98)		1		10.0 (4.0-23.2, 23)		1	
	Domiciles	13.2 (7.0-23.8, 76)		0.5 (0.2*-*1.1)	0.09~	8.0 (5.0-16.1, 10)		0.4 (0.2-1.1)	0.08
	Storerooms	6.0 (2.0-16.5, 49)		0.2 (0.1*-*0.6)	0.02*	5.0 (2.0-9.3, 3)		0.07 (0.02-0.2)	< 0.01**
	Kennels	15.0 (5.0-42.1, 13)		0.6 (0.1*-*2.4)	0.5	359.0 (10.0-708.2, 2)		21 (3.2-136)	0.001**
	Chickens coop	19.0 (13.0-29.6, 58)		0.7 (0.3-1.5)	0.4	11.0 (3.0-80.1, 10)		2.2 (0.8-6.2)	0.1
	Cow corrals	2.0 (0.0-12.5, 45)		0.1 (0.004*-*0.4)	0.01*	6.0 (-, 1)		0.04 (0.01-0.1)	< 0.001**
	Pig corrals	0.0 (0.0-14.2, 23)		-	-	-		-	-
	Rabbit hutches	40.0 (12.0-77.5, 5)		2.2 (0.3*-*1.4)	0.4	63.0 (60.0-66.2, 2)		4.4 (0.04-80)	0.3
	Others^c^	7.0 (2.0-21.3, 30)		0.2 (0.03*-*0.8)	0.06~	57.0 (1.0-114.2, 2)		0.6 (0.1-2.4)	0.5
Model 2	Intercept			0.07 (0.04-0.1)	< 0.001**			0.02 (0.004-0.1)	< 0.001**
	No. of chickens§		1	1.6 (1.2-2.2)	< 0.001**		1	2.2 (1.6-3.1)	< 0.001*
	No. of goats §		1	1.07 (1.03-1.1)	< 0.001**		1	1.1 (1.05-1.4)	< 0.001*
	No. of rabbits		0.7	1.1 (0.9-1.3)	0.1		< 0.1	1.04 (0.9-1.2)	0.6
	No. of cows and horses§t		0.5	0.2 (0.002-10.0)	0.4		1	0.03 (0.002-0.6)	0.02*
	No. of dogs and cats		0.4	1.2 (0.9-1.5)	0.2		1	6 (4.1-8.7)	< 0.001**
	Wall construction material		0.4				0.1		
	Wired metal, nylon, cloth,			1				1	
	wood without bark or wood planks								
	Bricks			3.4 (0.6-18)	0.1			0.2 (0.02-1.7)	0.1
	Mud			3.2 (1.1-9)	0.02*			2.7 (0.2-32)	0.5
	Branches, cane sticks			1.6 (0.7-3.6)	0.3			0.9 (0.4-2.2)	0.4

Infestation and abundance of *T. infestans* by ecotope (Model 1), and by type, number of host and construction materials in peridomestic sites (Model 2). House infestation was analysed by logistic regressions and bug abundance by negative binomial regressions. The odds ratio (OR) or incidence rate-ratio (IRR), their confidence intervals (CI_95_), relative importance (RI) and probability (p) are reported for each model. *: 0.01 ≤ p < 0.05, ~ p = 0.05 - 0.1; **: p < 0.01; § re-scaled variable to tens of hosts; a: infestation was determined by the finding of at least one live bug by timed-manual searches; b: bug abundance was calculated as the number of live bugs collected per 15 min-person among houses found infested by timed-manual searches; c: others: Galleries made of dry branches, piles of sticks, bricks, reeds and mud ovens, which appeared in low frequency.

**Fig. 2:**
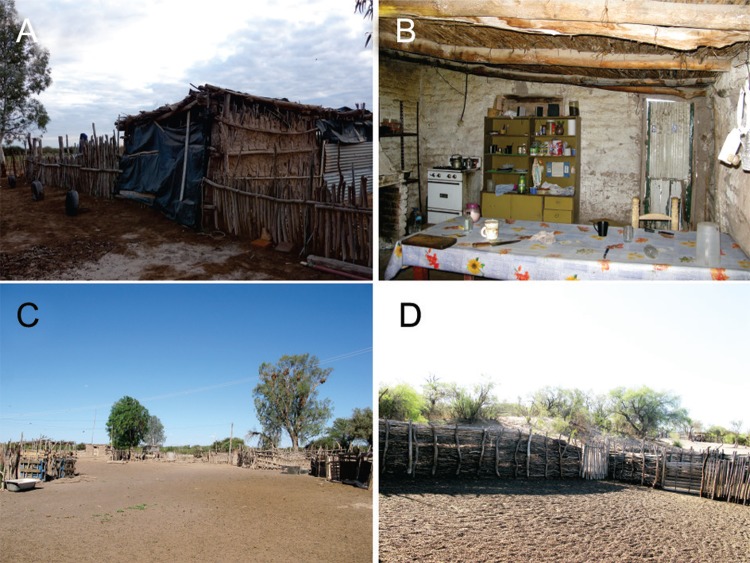
: typical rural houses and peridomestic structures in the La Asunción and San José Districts, Mendoza, Argentina. (A) Domicile made with mud and cane. (B) Interior of a domicile with a cane roof. (C) Goat corrals and chicken coops. (D) Typical goat corral with walls of stacked branches.

*House characteristics and socio-demographic surveys* - The 76 study houses were dispersed among a sandy area with steep dunes and difficult access; the average distance between houses was 10 km (Figs. 1-2). The houses were at an average distance of 32 ± 16 m from the nearest sylvatic habitat. A total of 141 inhabitants were registered in 76 houses [median household size = 3; interquartile range (IQR) = 1.5-4.0]. The interviewed householders were mostly adult men (77%; median age = 53; IQR = 31-66), and the remaining interviewees were women with a median age of 57 years (IQR = 31-69). Most (88%) the interviewees were born in the region, and the median length of residence was 19 years (IQR = 3-35); 53% of houses were inhabited only by people more than 15 years of age. The remaining households included children (median = 2, IQR = 1-3) who attended the rural school in periods of 15 consecutive days and then returned to their homes for a week.

A summary of the housing and socio-demographic characteristics of the study population is shown in Table I. Most houses were large (> 80 m^2^) and had mud walls and roofs made of dry branches, canes and mud with access to electricity through the electricity grid or solar panels.

**TABLE I:** Household socio-demographic and environmental characteristics and their associations with the infestation by *Triatoma infestans* in 76 domiciles in the La Asunción and San José Districts, Mendoza, May 2013

Variable		% of households (nº of houses)†	Domestic infestation	OR (CI)	p

			prevalence (CI)		
Presence of animals sleeping indoors					
Dogs and cats	No	64.5 (49)	12.2 (5.3-23.5)	1	
	Yes	32.9 (25)	16.0 (5.7-33.7)	1.4 (0.4-5.2)	0.61
Chickens	No	90.8 (69)	11.6 (5.6-20.7)	1	
	Yes	6.6 (5)	40.0 (9.4-79.1)	5.2 (0.9- 30.5)	0.07
Use of domestic insecticides					
	No	7.9 (6)	16.7 (1.9-55.8)	1	
	Yes	90.8 (69)	13.0 (6,7-22,5)	0.6 (0.1-4.0)	0.58
Type	Low concentration	92.8 (64)	12.5 (6.1-22.0)	0,4 (0,1-2,7)	0.32
	Deltamethrin	1.45 (1)	0	0.8 (0.02-32.4)	0.90
	Others^◊^	5.8 (4)	25.0 (2.9-71.6)	1	
Where	Domicile	95.3(61)	14.75 (7.6-25.2)	0.5 (0.2-14.4)	0.72
	Peridomicile	1.6 (1)	0	1	
	Both	3.1 (2)	0	0.6 (0.1-49.5)	0.82
Window screen use					
	No	44.7 (34)	20.6 (9.7-36.2)	1	
	Yes	53.9 (41)	7.3 (2.1-18.3)	0.3 (0.9-1.3)	0.11
Light source					
	Absent	1.3 (1)	0	-	
	Electricity	30.3 (23)	4.3 (0.5-18.6)	1	
	Solar panel	65.8 (50)	18.0 (9.3-30.3)	4.8 (0.6-40.6)	
Wall building material					
	Mud	17.1 (13)	0 (-)	1.3 (0.3-5.3)	0.69
	Bricks	28.9 (22)	13.6 (4.0-32.1)	1	
	Mixed	50.0 (38)	18.4 (8.6-32.8)	0.2 (0.01-4.3)	0.31
	No data	4.0 (3)	0 (-)	0.8 (0.03-19.3)	0.89
Roof building materials					
	Branches-canes-mud	17.1 (13)	15.4 (3.3-40.9)	6.4 (0.4-118.2)	0.21
	Metal-cement-wood	18.4 (14)	0 (-)	1	
	Mixed	60.5 (46)	17.4 (8.6-30.2)	6.3 (0.3-144.7)	0.25
	No data	4.0 (3)	0 (-)	4.1 (0.1-247.5)	0.50
Cracked walls					
	1-2^*^	38.1 (24)	8.3 (1.8-24.1)	1	
	3	54.0 (34)	14.7 (5.8-29.3)	1.7 (0.3-8.3)	0.52
	4	0 (0)	0	0	
	5	7.9 (5)	40.0 (9.4-79.1)	6.4 (0.3-0.4)	0.08

The Odds Ratio (OR), its confidence intervals (CI) and significance (p) obtained from the Firth penalised logistic regression univariate analysis of domestic infestation are presented. †: the total number of houses for each variable may be less than 76 owing to missing data; ◊: disinfectants such as chlorine or creolin; *: cracked walls classified from 1-5, where one represents few cracks and five abundant.

**Fig. 3:**
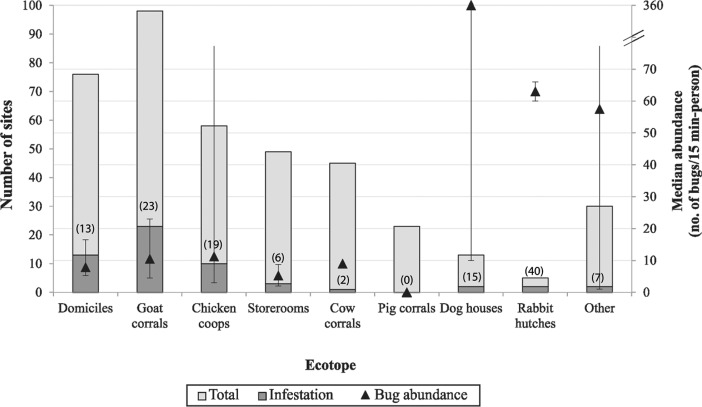
: number of infested and noninfested sites and the median abundance of *Triatoma infestans* by ecotope. Bars indicate the number of infested and noninfested sites; numbers between parentheses indicate the percentage of infestation by ecotope. Triangles indicate the median abundance of *T. infestans* by ecotope. Whiskers for bug abundance indicate the interquartile range. Others: pile of bricks, sticks, canes, ecotopes with no animal host associated, ovens.

Most households (93.4%) had at least one peridomestic structure. A total of 321 peridomestic sites were registered and mainly included goat corrals, chicken coops, storerooms and cow corrals (Fig. 3). Other habitats, such as pig corrals, dog kennels and rabbit hutches, were less frequent. Habitats such as galleries made of dry branches, piles of sticks, bricks, reeds and mud ovens were rare (each < 5%). The predominant building materials in the peridomestic sites were sticks (30%), cane or tree branches (26%), wood (13%) and wire (11%). Goat corrals were large and mainly made of sticks, canes and dry branches. Most chicken coops were built of dry branches and canes (41%) and wire-metal sheets (22%). Storerooms had mud or adobe walls (33%) and were less frequently made of cane and dry branches (20%).

Most households raised goats (86.3%; median household abundance = 170; IQR = 70-250) for commercial purposes and consumption (i.e., main economic activity). Poultry (in 55.7% of households), cows (22.2%), and pigs (21.1%) were raised for self-consumption and horses (37.7%) for transportation. The goat-equivalent index was relatively high (median = 167.7; IQR = 64.0-259.3).

*Domestic infestation and risk factors* - The domestic infestation was 13.2%, and seven of 10 houses with infested domiciles also had a concurrent peridomestic infestation. The median abundances of *T. infestans* in domestic (median = 7, IQR = 3-11) and peridomestic habitats (median = 7, IQR = 2-26) were similar. Domestic infestation rates increased with cracked walls and the presence of dry branches-canes-mud roofs and did not vary substantially with wall materials (mud or bricks) (Table I). Householders reportedly applied insecticides mainly in domiciles (87.1%), and window screens were present in more than half of the houses (53.9%). Domiciles that used insecticides and window screens had lower infestation rates, although these differences were not statistically significant (Table I).

The reported presence of domestic animals in domiciles was infrequent: 64.5% of households reported no dogs or cats residing indoors, and 90.8% reported no chickens nesting indoors. However, the infestation rate increased with the increasing presence of chickens in domiciles, although these differences were not statistically significant (Table I).

*Peridomestic infestation and risk factors* - Peridomestic infestation statistically differed among structures (χ^2^ = 24.2, df = 7, p = 0.001): goat corrals and rabbit hutches were frequently infested, followed by chicken coops and kennels (Table II, Fig. 3). Storerooms, cow corrals and other peridomestic structures (piled materials, ovens, etc.) were rarely infested (Table II, Fig. 3). When assessing the risk of infestation among ecotopes, only storerooms and cow corrals had significantly lower risk of being infested than did goat corrals, which were used as the reference category (Table II). Moreover, the risk of domestic infestation was lower than in goat corrals, although the difference was marginally significant (Table II). The effect of a house compound as a random variable was virtually nil, and no difference was found between models including a house as a random variable or not (Log-likelihood ratio test, p = 1), indicating that the effect of the ecotopes may have already accounted for the variation in the risk of infestation among houses. Both models were significantly different from the null model (Likelihood ratio test, p < 0.001); the area under the ROC curve was 0.7, with a sensitivity of 0.7 and specificity of 0.6, using an optimal threshold value of 0.14. The H-index was 0.12.

The relative abundance of *T. infestans* was also lower in storerooms and cow corrals than in goat corrals, whereas kennels had significantly higher bug abundance, which was greatly influenced by one site that harboured more than 700 bugs (Table II). No differences in bug abundance were found among other peridomestic ecotopes and domiciles. The bug abundance model significantly differed from the null model (Likelihood ratio test, p < 0.01). However, a large variability in bug abundance was observed among sites within ecotopes (Fig. 3), and no differences in bug abundance were found among ecotopes when only the infested sites were compared (Kruskal-Wallis test, p = 0.57). When removing the outlier value (700 bugs found in a kennel), the relative bug abundance of kennels decreased from 21 (CI = 3.2-136) to 0.01 (0.01-0.9), and it was significantly lower than bug abundance in goat corrals (p = 0.04). However, this removal had no effect on the remaining categories. The model considering the house as a random variable also showed no differences with the model that only considered the fixed-effects variables (Log-likelihood ratio test, p = 1). Lastly, a Poisson regression model was also evaluated and compared to the negative binomial regression model presented in Table II, but the latter presented a better fit with the data (AIC_Poisson_ = 8773, df = 9 vs. AIC_NegB_ = 555, df = 10), which confirmed the overdispersion of bug abundance among sites.

Infestation increased from 0 to 20.2% with increasing refuge availability (χ^2^ = 10.1, df = 4, p = 0.04). Overall, 39.2% of peridomestic sites were assigned to maximum refuge availability, and only 6.5% were assigned to the minimal level. Among habitats with top refuge availability (n = 119), goat corrals were the most frequent (40.3%) followed by chicken coops (16.8%) and storerooms (14.3%). Habitats with minimum refuge availability mainly included cow corrals (47.4%). Dry branches, canes and sticks were the main construction materials in all ecotopes (61%) and were almost the only material found in corrals (60-79%); hence, no clear association was found between construction materials and ecotopes. The presence and number of hosts varied among ecotopes: cows, horses, goats, pigs and rabbits reportedly occurred only at their respective corrals, whereas chickens, dogs and cats occurred in more than one ecotope.

When assessing the effects of type and number of hosts and construction materials on the risk of peridomestic infestation, the risk increased significantly with the number of goats and chickens. An increase of 10 chickens increased the risk of infestation by 90%, whereas an increase of 10 goats only increased the risk of infestation by 6% (Table II). Pigs were not included in this analysis, because no pig corral was found to be infested, and these hosts reportedly occurred only in this ecotope. Regarding construction materials, the presence of mud or adobe walls was significantly and positively associated with infestation (Table II), unlike bricks, dry branches and cane sticks. This model had an area under the ROC curve of 0.81, with a sensitivity of 0.79 and specificity of 0.74, using an optimal threshold value of 0.12, and presented a good fit to the data (Hosmer-Lemeshow test, p = 0.2). The H-index value was 0.37. Its better classification performance compared with the infestation model considering only ecotope as an independent variable (AUC = 0.7, H-index = 0.12) suggests that the risk of infestation in a given peridomestic ecotope (Table II, Model 1) was mainly determined by the abundance and type of hosts available and, second, by construction materials (Table II, Model 2).

Bug abundance increased with the abundance of dogs or cats, chickens and goats and decreased with the number of cows and horses, whereas construction materials did not have any effect on bug abundance (Table II). After removing the kennel that harboured 700 bugs from the model (Table II, Model 2), the estimated incidence rate-ratio of the abundance of dogs and cats changed from 6 (CI = 4.1-8.7) to 1.1 (CI = 0.7-1.5), and the effect became non-significant (p = 0.6). The estimated incidence rate-ratio of the abundance of other hosts remained unchanged, but the estimated effects of construction materials in walls also changed: mud or adobe walls were inversely associated with bug abundance (IRR = 0.3, CI = 0. 08-0.9, p = 0. 04).

The VIF estimated for the variables included in the multivariate analysis of peridomiciliary infestation and bug abundance showed no evidence of multicollinearity; all variables showed a VIF < 2.

*Infection with T. cruzi* - Table III shows that the overall prevalence of *T. cruzi* infection in *T. infestans* was 4.1% (95%CI = 2-7%; n = 412). Infection among domestic bugs (n = 59) was 10.2% (95% CI = 4-21%) and came from three houses, whereas the rate of peridomestic bug infection (n = 348) was 3.2% (95% CI = 2-3%). The prevalence of *T. cruzi* infection among domestic and peridomestic bugs was significantly different (χ^2^ = 6.2, df = 1, p = 0.013). The infected peridomestic triatomines were collected from four goat corrals, two kennels and two storerooms in eight houses.

**TABLE III:** Prevalence of *Trypanosoma cruzi* infection in *Triatoma infestans* collected in domestic and peridomestic habitats from 76 domiciles in the La Asunción and San José Districts, Mendoza, May 2013

Ecotope	Stage	Bugs collected (nº)	Bugs examined by kDNA-PCR (nº)	Infected (%)
Domestic	First-second	7	0	0.0
	Third-fifth	56	32	9.4
	Adult	51	27	11.1
Peridomestic	First-second	237	0	0.0
	Third-fifth	894	234	4.3
	Adult	441	119	0.8

Total		1,686	412	4.1

kDNA-PCR: kinetoplast minicircle-polymerase chain reaction.

**Fig. 4:**
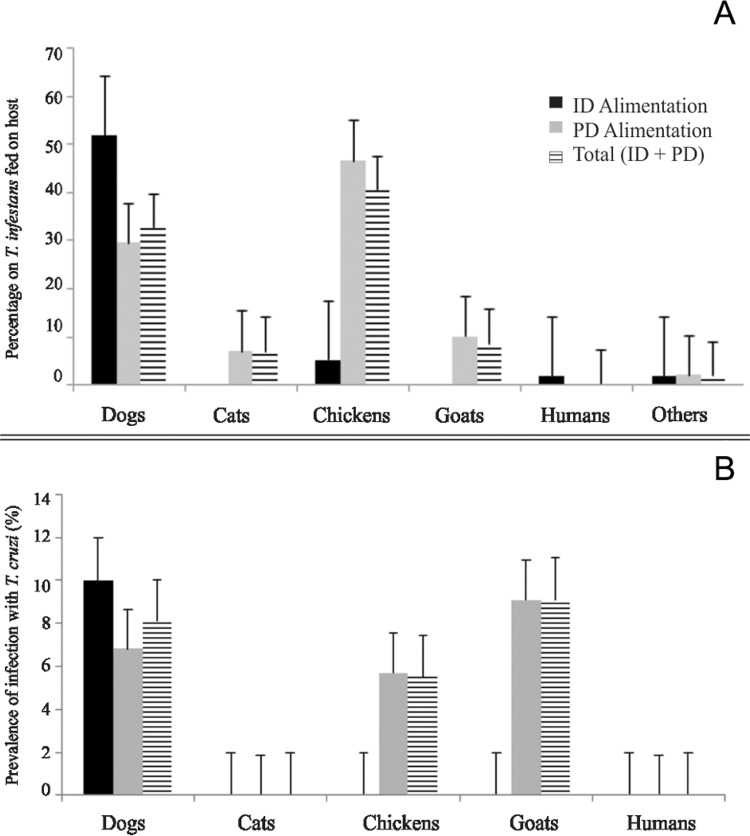
: host-feeding patterns of *Triatoma infestans* and prevalence of infection with *Trypanosoma cruzi* in *T. infestans*. Bugs collected in domestic (ID) and peridomestic (PD) sites (A) and the prevalence of infection according to bloodmeal sources in domestic and peridomestic ecotopes (B). Others: pigs, rabbits, guinea pigs and rats.

*Host-feeding sources* - At least one bloodmeal source was identified in 59 (58%) domestic bugs and in 356 (81%) peridomestic bugs. These differences were statistically significant (Fisher’s exact test, p < 0.01). Dogs were the main bloodmeal source in domiciles (51.7%; CI = 38*-*65%) followed by chickens (5.2%; CI = 1-14%) (Fig. 4A). Only one sample was reactive for human blood, and no blood meal from cats was detected. The main bloodmeal sources in peridomestic sites were chickens (46.5%, CI = 41-52%), dogs (27.5%, CI = 23-32%) and goats (10.1%, CI = 7*-*14%) (Fig. 4A). Blood meals on cats (2.0%, CI = 1-4%), cavies (1.1%, CI = 1-3%), rabbits, pigs and murid rodents (0.3%, CI = 0.01-2%) were rare.

*T. cruzi* infection in domestic *T. infestans* occurred exclusively in bugs that fed on dogs (10.0%; CI = 2*-*27%), and the human-fed bug was not infected. In peridomestic habitats, similar infection rates were detected in bugs that fed on goats (9.1%; CI = 2-24%), dogs (6.8%; CI = 1-19%) and chickens (5.7%; CI = 2-13%) (Fig. 4B).

## DISCUSSION

Our results document the occurrence of domestic and peridomestic infestation with *T. infestans* in northern Mendoza. Unlike other endemic rural areas, baseline domestic infestation was much lower than expected given that insecticide spraying campaigns had historically been very sporadic. Nonetheless, our results document the occurrence of *T. infestans* infected with *T. cruzi* in human sleeping quarters and in peridomestic sites. Bloodmeal identification tests revealed that the bugs mainly fed on dogs, chickens and goats. The greatest risk of infestation was mainly associated with goat corrals, which provided appropriate refuges and hosts for *T. infestans* bugs.

The socio-demographic analysis of local households showed an aged population with a long-standing settlement history in precarious houses made of adobe, sticks, dry branches and canes and whose main economic activity was goat husbandry. Young people emigrated to neighbouring irrigated areas after finishing secondary school in search of better life prospects. Water is very scarce in arid Mendoza and is mainly accessible in the oases where grapevines and fruit trees are cultivated. Goat husbandry is the main economic activity in the vast sandbanks desert, such as that included in our study area. Mendoza province ranks third in goat production at a national level, and Lavalle Department is the second largest producer in the province (Abraham & Torres 2014). This activity occurred in 86% of the households and was the basis of the local economy. The goat-equivalent index was 2.4-times higher than that recorded elsewhere in the Argentine Chaco (Gaspe et al. 2015). Poultry and cows were used for consumption and/or commercialisation, while horses were used for transportation.

*Determinants of domestic infestation* - We expected to find a higher prevalence of domestic infestation in Lavalle based on favourable conditions for *T. infestans* (i.e., traditionally endemic area, last insecticide spraying campaign occurring 2-10 years before, precarious rural housing and other socio-demographic factors). However, the observed infestation rates were much lower than elsewhere in northern Argentina (Gurevitz et al. 2011, Gaspe et al. 2015), and domestic infestation mainly occurred in houses with peridomestic infestation (Gurevitz et al. 2011). In addition, some domicile characteristics and household practices were related to higher infestation, although not significantly so: roofs made of dry branches, cane and mud, absence of window screens, cracked walls, and the indoor presence of dogs, cats and chickens. Domestic infestation was lower in households that reportedly applied domestic insecticides, although the difference was not significant. Although few domiciles had dogs, cats or chickens resting indoors, domestic infestation increased (non-significantly) with their presence, especially in the case of indoor chickens, as reported in other regions (Gurevitz et al. 2011). The frequent domestic use of insecticides and window screening may explain, at least in part, why house infestation with *T. infestans* was much lower than expected. The large abundance of domestic flies during the hot season may also be related to preventive practices. The use of electricity and proximity to wild habitats may favour the invasion of triatomines from wild or peridomestic foci into domiciles, especially among houses that lack window screens (Waleckx et al. 2015). Active dispersal of nymphs and adults of *T. infestans* and other triatomines were reported in different areas of Argentina (Abrahan et al. 2016).

*Determinants of peridomestic infestation* - Peridomestic ecotopes had an increased risk of infestation, although both infestation and bug abundance differed among particular structures. Rabbit hutches were frequently infested, although the hutches were rare. Second, goat corrals were infested structures, which coincided with the ready availability of appropriate refuges for *T. infestans*, followed by chicken coops. Conversely, cow corrals and storerooms showed a lower risk of infestation than did goat corrals. Peridomestic structures were important sources of reinfestation in both natural (Gurevitz et al. 2011) and experimental conditions (Gorla & Schofield 1989) elsewhere in the Argentine Chaco. A very large colony of *T. infestans* was found in a kennel with four dogs located 30 m from the nearest human sleeping quarters and 100 m from the nearest house. Given that *T. cruzi*-infected bugs were collected there, this kennel most likely acted as a source of dispersing infected triatomines.

Multivariate analysis of the determinants of peridomestic infestation revealed the significant effects of the number of goats and chickens, suggesting that goat corrals and chicken coops were more likely to be infested and harboured larger numbers of *T. infestans* than other ecotopes. We also identified that mud increased the risk of peridomestic infestation regardless of the type and number of local hosts.

Our results emphasize the relative importance of specific peridomestic structures (i.e., rabbit hutches, goat corrals, chicken coops, dog kennels) for house infestation with *T. infestans*. A large fraction of houses with infested domiciles also harboured a peridomestic focus, mainly in goat corrals. Recently, two alternative control methods to reduce domestic triatomine populations have been trailed in Argentina: a motorised vehicle-mounted sprayer for insecticide application, which restricted end-point infestations to peridomiciles (Carbajal-de-la-Fuente et al. 2017), and modification of traditional goat corrals, which increased goat productivity and likely enhanced the detectability of low-density infestations (Gorla et al. 2013). A combination of environmental management and improved chemical vector control may be needed to suppress peridomestic infestations. Considering the economic importance of goat corrals, community participation is expected to play a crucial role if environmental management measures are to be introduced.

*T. cruzi infection and host-feeding sources* - The overall prevalence of *T. cruzi* infection (4.1%) in *T. infestans* was relatively low, but the domestic infection rate was rather high. The domestic abundance of infected fifth-instar nymphs and adult bugs suggested the occurrence of parasite transmission indoors, many of which had recently fed on dogs. Although the infected bugs may have acquired the infection from a previous blood meal, dogs and cats frequently are the main domestic reservoir hosts of *T. cruzi* and a risk factor for transmission in Argentina and elsewhere (Gürtler & Cardinal 2015). Multivariate analyses of peridomestic infestation and bug abundance showed that chickens, goats and dogs or cats represented the main hosts and bloodmeal sources of *T. infestans* (e.g., Gurevitz et al. 2011). High host mobility between sites likely contributed to persisting site infestation and infection. In this study, human blood meals were infrequent, which suggested that human-vector contact was very limited at the time of our survey.

Our study had some limitations. The survey was conducted as part of a randomised intervention trial that sought to evaluate the impacts and performance of various insecticide spraying operations in harsh terrain. The results were obtained from a limited number of infested houses that had been previously identified by the Chagas vector control programme; therefore, the study houses were a selected sample, and this may have reduced the power of significance tests. Logistic restrictions dictated that vector surveys were conducted in mid-fall, when temperatures averaged 20.4 ± 0.9ºC and possibly the abundance of *T. infestans* and/or the probability of bug detection by timed-manual searches were decreasing. The physical complexity of goat corrals may also reduce the detectability of triatomines by timed-manual searches. The low domestic infestation combined with a rather small sample size also limited our ability to identify factors associated with house infestation. Blood-feeding patterns determined by direct ELISA may detect blood meals that have occurred within the previous two or three months, depending on site-specific temperatures and other factors. Therefore, bloodmeal results correspond to a rather undefined time window.

The occurrence of domestic and peridomestic infestation with *T. infestans* and *T. cruzi* infection supports the hypothesis that vector-borne transmission still occurs in northern Mendoza. Although vector control actions in the affected region have increased substantially since 2008, the results of the present study justify the implementation of additional control actions combined with sustained vector surveillance. Historically, the problem of Chagas disease and vector control has been addressed mainly from a biomedical perspective. Incorporating the socio-cultural and political dimensions of the problem and recognising the equivalent importance of these four interdependent dimensions is crucial (Sanmartino et al. 2016). The affected communities should be recognised as key stakeholders and included in decisions regarding how to better control house infestation and conduct vector surveillance. Their contributions to better husbandry practices and the construction and maintenance of peridomestic structures, especially goat corrals, may lead to improved vector control strategies.
